# Understanding and Advancing Wound Healing in the Era of Multi-Omic Technology

**DOI:** 10.3390/bioengineering13010051

**Published:** 2025-12-31

**Authors:** Serena L. Jing, Elijah J. Suh, Kelly X. Huang, Michelle F. Griffin, Derrick C. Wan, Michael T. Longaker

**Affiliations:** Department of Surgery, Division of Plastic and Reconstructive Surgery, Stanford University School of Medicine, Stanford, CA 94305, USAmgriff12@stanford.edu (M.F.G.);

**Keywords:** wound healing, fibrosis, multi-omics, artificial intelligence

## Abstract

Wound healing is a complex, multi-phase process requiring coordinated interactions among diverse cell types and molecular pathways to restore tissue integrity. Dysregulation can lead to chronic non-healing wounds or excessive scarring, posing major clinical and economic burdens. Single-omics interrogate individual molecular layers, such as the genome, transcriptome, proteome, metabolome, or epigenome, and have revealed key cellular players, but provide a limited view of dynamic wound repair. Single-cell technologies provide higher resolution to single-omic data by resolving cell-type and state-specific heterogeneity, enabling precise characterization of cellular populations. Multi-omics integrates multiple molecular layers at single-cell resolution, reconstructing regulatory networks, epigenetic landscapes, and cell–cell interactions underlying healing outcomes. Recent advances in single-cell and spatial multi-omics have revealed fibroblast subpopulations with distinct fibrotic or regenerative roles and immune–epithelial interactions critical for re-epithelialization. Integration with computational tools and artificial intelligence (AI) continues to reveal cellular interactions, predict healing outcomes, and guide development of personalized therapies. Despite technical and translational challenges, including data integration and cost, multi-omics are increasingly shaping the future of precision wound care. This review highlights how multi-omics is redefining understanding of wound biology and fibrosis and explores emerging applications such as smart biosensors and predictive models with potential to transform wound care.

## 1. Introduction

Wound healing is a complex biological process requiring the precise coordination of molecular pathways to progress the wound from initial injury to re-epithelialization and remodeling. Under normal conditions, this process results in fibrosis, which manifests as visible scars. However, comorbidities such as diabetes and vascular diseases can disrupt normal wound repair, leading instead to chronic, non-healing wounds. Conversely, excessive healing responses can produce hypertrophic scars or keloids. These pathological outcomes represent a substantial healthcare burden—in the United States alone, chronic nonhealing wounds account for approximately $50 billion annually [1], while scars resulting from surgical incisions and trauma contribute nearly $20 billion in additional healthcare costs [2].

Despite recent advances in molecular research that have provided valuable insights into wound healing, identifying a universal therapeutic target capable of promoting a regenerative wound healing response remains elusive [3]. A deeper understanding of the molecular mechanisms underlying wound healing is therefore essential—not only to bridge the translational gap in reducing visible scarring, but also to effectively address the clinical challenges posed by both impaired and excessive healing.

Multiple cell types, such as fibroblasts, macrophages, and endothelial cells, contribute unique and essential roles to each phase of wound healing [4]. Traditional research approaches have largely relied on single-omics methodologies to study these cells in isolation, which has yielded important insights into how they function individually, and their plasticity. However, they fail to capture the full complexity of wound healing, which not only arises from the activity of individual cell types but also from their dynamic interactions with each other and with the surrounding microenvironment, including mechanical, biochemical, and spatial cues. In fact, the paucity of translational advancements in clinical wound healing underscores the inherent limitations of relying exclusively on single-omics or any single research approach when investigating the intricate processes underlying wound healing and fibrosis [5].

Multi-omics, which integrates single-omic technologies, provides a comprehensive view of cellular processes and the diverse factors shaping cell behavior. By combining multiple-omic layers, such as genomics, transcriptomics, epigenetics, proteomics, metabolomics, and spatial transcriptomics, causal relationships among molecular components may be better understood. These integrative methods have significantly advanced our ability to dissect complex diseases and biological processes, offering a holistic perspective on molecular changes during tissue repair and providing deeper insights into the underlying mechanisms driving disparate healing outcomes [5].

This review explores the current state and future promise of multi-omic technologies in advancing our knowledge of wound healing and fibrosis and highlights their potential in the development of innovative and personalized treatments to achieve the ideal wound healing response—one that avoids excessive or insufficient healing and minimizes fibrosis.

## 2. Review

### 2.1. Fundamentals of Wound Healing

Wound healing is a tightly regulated process that progresses through four overlapping phases: hemostasis, inflammation, proliferation, and remodeling. Here, we highlight some of the dominant cell types responsible for progression through each phase.

#### 2.1.1. Hemostasis

Immediately after injury, platelets adhere to exposed collagen, initiating clot formation and the release of growth factors like platelet-derived growth factor (PDGF) and transforming growth factor beta (TGF-β) [4]. These growth factors help activate mesenchymal cells, such as those in the smooth muscle of the vessel walls, leading to contraction to reduce bleeding. Notably, however, while platelets are vital for clot formation, mouse studies have indicated that their depletion does not critically impair wound healing, as growth factor release, rate of wound epithelialization, collagen synthesis, and angiogenesis remain largely preserved [6].

#### 2.1.2. Inflammation

The inflammatory phase of wound healing begins within the first 24 h of injury, marked by the rapid infiltration of neutrophils and macrophages into the wound site. Neutrophils, which comprise more than 50% of the wound cell population on the first day post-injury, play a critical role by destroying pathogens through phagocytosis, oxidative bursts, and toxic granule release [7]. Macrophages are another critical immune cell type in this phase, responsible for clearing dead cells and secreting factors that promote angiogenesis, granulation tissue formation, and collagen deposition [4]. Animal studies have demonstrated that macrophage depletion delays wound closure by disrupting these critical processes, highlighting their essential role in proper wound resolution and progression to the reparative stages [8].

#### 2.1.3. Proliferation

The proliferative phase of wound healing is characterized by granulation tissue formation and neovascularization. Fibroblasts are central to granulation tissue formation [9]. They synthesize extracellular matrix (ECM) components and contract the wound to facilitate closure. Fibroblasts exhibit significant heterogeneity, influenced by their anatomical location, embryonic origin, and activation state [10]. This heterogeneity influences healing outcomes, with certain subsets driving scar formation through excessive ECM deposition. As fibroblasts are secreting collagenous ECM, keratinocytes begin to migrate across the surface of the wound [4].

Endothelial cells are essential for neovascularization, responding to pro-angiogenic signals such as vascular endothelial growth factor (VEGF), PDGF, and fibroblast growth factor (FGF) to form new capillaries. This newly established vasculature delivers critical nutrients and oxygen to the wound bed, facilitating tissue repair [4].

#### 2.1.4. Remodeling

The remodeling phase represents the final phase of wound healing, extending for months or even years after closure [4,11]. During this stage, granulation tissue, rich in collagen III, matures into stable scar tissue composed of collagen I. This process is mediated by matrix metalloproteases (MMPs), which degrade components of the ECM, and tissue inhibitors of metalloproteases (TIMPs), which regulate this degradation [12]. A balance between MMPs and TIMPs is important as disruption in this balance can lead to underhealing or overhealing wounds [4,13]. Myofibroblasts, which produce ECM and contractile elements, normally undergo apoptosis during this phase. Their persistence can lead to excess matrix deposition and fibrosis [14].

#### 2.1.5. Aberrant Wound Healing: Overhealing and Underhealing Wounds

Disruptions in this process can result in chronic wounds or excessive scarring, manifesting as hypertrophic scars and keloids. It is clear that no one cell type drives this process; rather, it depends on the coordinated actions of diverse cell populations across various stages of repair. Understanding the factors that drive the behaviors of these key cells, initiate the transitions between the stages of repair, and govern cell-to-cell interactions within the spatial and temporal dynamics of the wound microenvironment therefore becomes essential.

### 2.2. Overview of Multi-Omic Technologies

The emergence of multi-omic technologies has ushered in a transformative era in biomedical research, enabling unprecedented insights into the molecular mechanisms underlying complex biological processes and disease states.

The completion of the Human Genome Project in 2003, which mapped the entire human genome, marked a pivotal moment in biomedical research [15]. It served as the catalyst for the development of sequencing technologies, such as next-generation sequencing and mass spectrometry, significantly reducing the cost and time of sequencing [15,16]. These advancements laid the foundation for the emergence of multi-omic technologies, enabling comprehensive profiling of the genome, proteome, transcriptome, epigenome, and metabolome [17].

#### 2.2.1. Genomics

Genomics examines an organism’s complete set of DNA and has demonstrated significant relevance in wound healing by uncovering genetic factors that influence wound repair, fibrosis, and contribute to the susceptibility of developing hypertrophic scars or chronic wounds [17]. During the inflammatory phase of wound healing for instance, genome-wide association studies (GWAS) have linked variants in the genes Talin-2 (TLN2) and Zinc finger protein 521 (ZNF521) to an increased abundance of pathogens like *Pseudomonas aeruginosa* [18]. In *Pseudomonas aeruginosa* infected wounds, wound microbial diversity decreases, which was also further correlated with delayed healing. In a separate GWAS of hypertrophic scars, variants such as the rs11136645 in the CUB and Sushi multiple domains 1 gene (CSMD1), were also identified to be associated with decreased scar height, implicating the gene as a potential regulator of post-burn wound repair [19]. Although the functional consequences of this variant remain to be fully understood, CSMD1 has been suggested to also play a role in the inflammatory phase of wound healing, serving as an inhibitor of the classical and lectin complement pathways [20]. Attenuation of complement-mediated inflammation through enhanced CSMD1 activity could dampen the early pro-inflammatory response, thereby mitigating downstream proliferative signaling of fibroblasts, associated with the formation of hypertrophic scars. In addition, CSMD1 has been linked to the regulation of TGF-β1 and SMAD signaling, central drivers of fibroblast activation, myofibroblast activity, and consequent excessive ECM deposition during the proliferative and remodeling phases of wound healing [21]. Thus, it has been suggested that CSMD1 may exert anti-fibrotic effects by curbing TGF-β-driven collagen production and myofibroblast activity, both of which are key features of pathological scar formation.

These genetic variants, identified through genomic studies, have the potential to serve as biomarkers to help stratify patients based on their susceptibility to certain pathogens, and serve as therapeutic targets for fibrotic skin disorders.

#### 2.2.2. Proteomics

Proteomics studies all proteins expressed in an organism. Unlike genomics, which provides a static blueprint, proteomics reveals dynamic changes in protein expression in response to cellular and environmental signals [22]. Technologies such as mass spectrometry and protein microarrays have enabled high-throughput analysis of thousands of proteins simultaneously. In wound exudates from chronic non-healing wounds, proteomic analyses have identified S100A9, a calcium binding inflammatory mediator, as significantly elevated compared to normal wound exudates. Its abundance in nonhealing wounds has suggested a persistent pro-inflammatory wound environment that fails to progress the wound to the reparative phases of healing [23]. During the proliferative phase, proteomic analyses have shown a clear enrichment of ECM proteins in healing wounds, including collagen I and III, which are key markers of granulation tissue maturation [24]. Notably, these collagens were absent in chronic wound exudates, suggesting a failure to initiate matrix synthesis and tissue remodeling. Additional proteins, like tetranectin and those from the serine protease inhibitor (SERPIN) family (SERPINA1, SERPINA3) were significantly upregulated in healing wounds, promoting plasminogen activation, matrix stability, and angiogenic balance, which are critical in the proliferative phase of wound healing [24]. In contrast, chronic wounds exhibited a dominance of provisional matrix proteins, like fibronectin and vitronectin, indicative of wounds stalled in the early proliferative phase.

Collectively, these findings provide a foundation for identifying surrogate biomarkers that can enhance our understanding of wound healing dynamics and improve the prediction of successful versus impaired healing outcomes.

#### 2.2.3. Transcriptomics

Transcriptomics provides the complete set of RNA transcripts produced within an organism in real-time, offering a dynamic view of gene expression in response to physiological or environmental changes. RNA sequencing (RNA-seq), microarray analysis, and single-cell RNA sequencing (scRNA-seq) are technologies that allow for the quantification of gene expression levels and identification of novel transcripts [25]. Transcriptomics can reveal temporal changes in gene expression driving inflammation, tissue regeneration, and fibrosis, providing insights into the molecular regulation of these processes. In keloids, transcriptomic analyses conducted through scRNA-seq have identified myofibroblasts as a dominant and aggressive population, characterized by upregulation of genes such as Yes-associated protein (YAP), Tafazzin (TAZ), Piezo-type mechanosensitive ion channel component 1 (Piezo1), Ras homolog family member A (RhoA), and Rho-associated protein kinase 2 (ROCK2) [26].

Transcriptomic profiling has also enhanced our understanding of the distinct phases of wound healing, uncovering specific gene expression signatures associated with hemostasis, inflammation, proliferation, and remodeling. While the early phases of hemostasis and inflammation exhibit substantial variability depending on tissue type and injury context, consistent transcriptional patterns begin to emerge during the proliferative and remodeling phases, where genes involved in extracellular matrix organization (collagen type I alpha 1 chain (COL1A1), MMP12, CD44), angiogenesis (VEGFA), and epithelial migration (CC Motif Chemokine Ligand 2 (CCL2)) become increasingly upregulated [27]. In MMP12 knockout models, a significant reduction in the fibrosis of lung and liver was observed, suggesting that targeting the activity of specific MMPs may be a viable approach to treating fibrosis in other organs [28]. Meta-analyses of wound transcriptomes have also observed the expression of different groups of immune response genes that are not solely limited to the inflammatory phase—instead they continue to remain active during the proliferation and remodeling phases of wound healing, where they likely contribute to tissue repair, resolution of inflammation, and matrix remodeling [27]. This preliminary finding underscores the significance of further understanding the immune-stromal crosstalk in orchestrating effective tissue regeneration.

#### 2.2.4. Epigenomics

Epigenomics examines changes in gene expression that do not result in alterations of the genetic code. Assay for Transposase-Accessible Chromatin Sequencing (ATAC-seq) is a high-throughput method that uses a Tn5 transposase to cut and tag regions of open chromatin, allowing genome-wide mapping of accessible regulatory elements such as promoters, enhancers, and transcription factor binding sites with relatively low cell input [29]. In the context of wound healing, ATAC-seq has been used to define cell type-specific regulomes in keratinocytes, fibroblasts, and immune cells, revealing distinct accessibility signatures and transcription factor networks that change between healthy, injured, or fibrotic skin [30,31]. For example, ATAC-seq of dermal fibroblasts from irradiated skin has revealed persistent chromatin accessibility at the wound-repair gene thrombospondin 1 (THBS1), creating an epigenetically primed fibroblast state that drives excessive THBS1 production and impaired healing- an effect shown to be reversible with THBS1 antibody blockade [32].

Techniques like Chromatin Immunoprecipitation Sequencing (ChIP-seq) also allow for the identification of regulatory elements that shape the transcriptional landscape of cells involved in wound healing [33]. For example, epigenomics has revealed Polycomb group (PcG) proteins, which are a family of epigenetic modifiers, to regulate the expression of genes during the repair process. Using ChIP-seq, loss of PcG-mediated gene silencing was shown to reactivate large sets of genes necessary for epithelial cell migration, proliferation, and tissue remodeling [34].

Furthermore, in the proliferative phase of wound healing, epigenetic regulation has been shown to be vital for keratinocyte proliferation and migration [35]. DNA methyltransferase 1 (DNMT1) is crucial for maintaining epidermal stem cell self-renewal, and its downregulation is associated with impaired re-epithelialization. DNMT1′s expression has been shown to increase during wound closure, and its silencing has been shown to lead to defects in keratinocyte differentiation and delayed epithelial repair [35]. During the remodeling phase, epigenomic mechanisms regulate fibroblast activity and ECM turnover. In keloids and other sclerotic disorders, fibroblasts exhibit global methylation changes, including hypermethylation of anti-fibrotic genes and hypomethylation of pro-fibrotic regulators like Runt-related transcription factor 2 (RUNX2) and alpha-smooth muscle actin (α-SMA) [35,36]. These alterations drive excessive collagen deposition and myofibroblast persistence, leading to pathologic scarring. Inhibition of DNA methyltransferases in fibrotic models has been shown to reduce ECM accumulation and partially reverse the fibrotic phenotype, suggesting therapeutic potential [37]. By elucidating how methylation patterns affect immune cell function, keratinocyte dynamics, and fibroblast activation, epigenomics may help to bridge the gap between molecular signaling and clinical outcomes such as chronic wounds or hypertrophic scars.

#### 2.2.5. Metabolomics

Metabolomics is the comprehensive study of small-molecule metabolites, such as lipids, amino acids, and other intermediates, and their biochemical activities within cells and tissues. Techniques like mass spectrometry and nuclear magnetic resonance (NMR) spectroscopy enable high-resolution identification and quantification of these metabolites [38].

In a novel multi-time point study of acute wounds, distinct metabolic profiles were observed at different stages of wound healing [39]. During the inflammatory phase, metabolites such as linolenic acid were significantly elevated and found to modulate immune responses by suppressing pro-inflammatory cytokines like Tumor necrosis factor alpha (TNF-α) and Interleukin-12 (IL-12). As the wound progressed to the proliferative phase of wound healing, metabolite levels associated with cell migration, angiogenesis, and ECM formation began to dominate. For example, D-(+)-galactose correlated with optical coherence tomography measures of ECM remodeling, reflecting its role in promoting granulation tissue formation. Glycerol, which was another metabolite measured to be significant during this phase, is known to support re-epithelialization by enhancing hydration, elasticity, and barrier repair [40]. During the remodeling phase, specific metabolites, such as 1,3-bis(1,1-dimethylethyl)benzene and other phenylpropane derivatives, were linked to tissue remodeling metrics, including collagen deposition and attenuation compensation [39]. These findings highlight specific metabolites involved in apoptosis and collagen organization, which play key roles during late-stage wound remodeling.

In another study of diabetic mouse wounds, 62 metabolites were identified to be significantly altered in response to injury. Among these metabolites were arginine, a precursor for the vasodilator nitrous oxide, and 3-nitrotyrosine, a marker of oxidative damage, further underscoring the potential use of biomarkers for wound diagnoses and prognoses to be identified [41].

**Figure 1:**
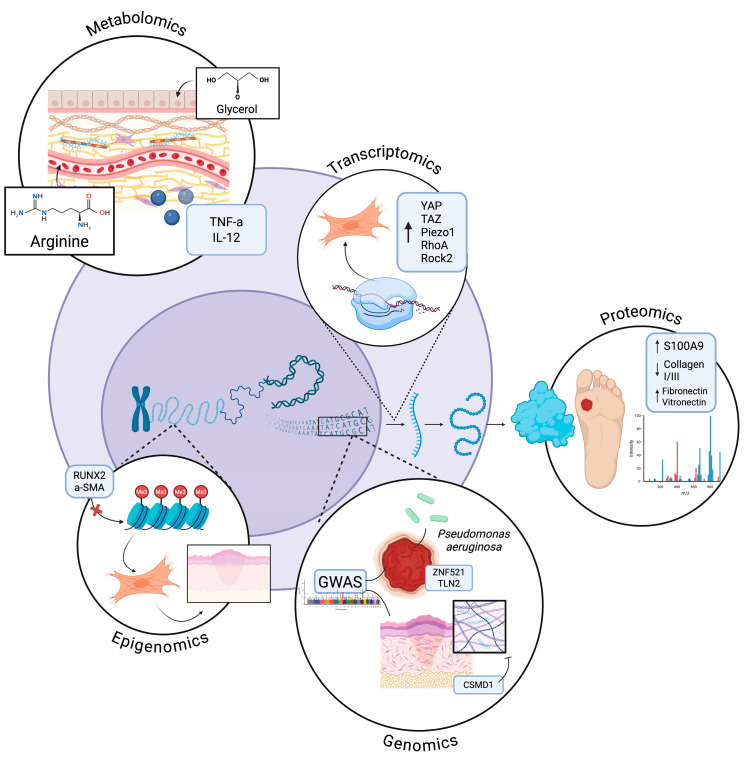
Cellular Integration of -Omic insights in Wound Healing. Depiction of how genomics, transcriptomics, proteomics, epigenomics, and metabolomics collectively regulate wound healing. Genomics identifies inherited risk variants affecting susceptibility to infection, inflammation, and fibrosis. Proteomics captures dynamic protein changes, including inflammatory mediators and ECM components. Transcriptomics reveals gene network activity across healing phases while epigenomics uncovers chromatin and methylation patterns controlling cell behavior. Metabolomics highlights small-molecule mediators that modulate inflammation, angiogenesis, and tissue repair. Collectively, these -omics insights illuminate the complex, multi-layered regulation of wound healing. Created in BioRender. Jing, S. (2025) https://BioRender.com/toglotk.

While each single-omics approach offers valuable insights into wound healing (Table 1), they ultimately fall short of capturing its full complexity. Genomics identifies inherited risk variants, such as TLN2, ZNF521, and CSMD1, that influence susceptibility to infection, inflammation, or fibrosis. However alone it is unable to establish causality or context-specific regulation. Proteomics captures dynamic protein expression changes, such as elevated S100A9 or altered ECM components like collagen I and III, but cannot explain how these are transcriptionally or epigenetically regulated. Transcriptomics reveals the activity of gene networks during each healing phase, such as YAP/TAZ upregulation in keloid fibroblasts or the continued expression of immune genes into the remodeling phase; however, it often misses upstream regulatory cues and post-transcriptional modifications. Epigenomics and metabolomics provide critical insight into chromatin dynamics and metabolic reprogramming that shape wound outcomes, such as DNMT1-mediated keratinocyte function or linolenic acid’s role in suppressing inflammation. However, these approaches often lack spatial and cell-type resolution. Ultimately, while each -omics layer offers a valuable lens, it is the integration of these multi-omic technologies that enables a systems-level understanding of wound healing, revealing cross-regulatory networks, cell–cell interactions, and temporally resolved pathways that drive successful or pathological repair (Figure 1).

### 2.3. Integration of Multi-Omic Data

**Figure 2:**
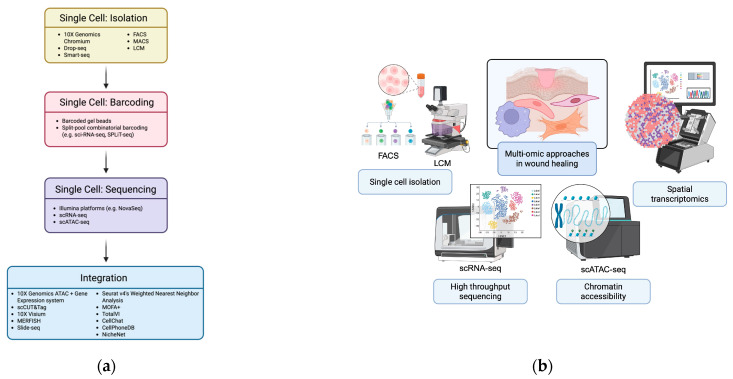
Integration of multi-omic approaches at single-cell resolution. (**a**) Workflow illustrating single-cell processing and multi-omic data integration. Cells are isolated using droplet-based platforms (10× Genomics Chromium, Drop-seq, Smart-seq, FACS, MACS, LCM) and barcoded via barcoded gel beads or split-pool combinatorial indexing. Sequencing (scRNA-seq, scATAC-seq) and multi-omic integration—through experimental methods (10× ATAC + Gene Expression, scCUT&Tag, spatial approaches) and computational tools (Seurat v4, MOFA+, TotalVI, CellChat, CellPhoneDB, NicheNet)—resolve cellular states, interactions, and signaling networks. Created in BioRender. Jing, S. (2025) https://BioRender.com/ts8rx0p (**b**) Schematic overview of single-cell technologies showing how these approaches simultaneously capture genomic, transcriptomic, epigenomic, and proteomic data, linking the workflow in (**a**) to comprehensive, multi-dimensional cellular profiling. Created in BioRender. Huang, K. (2025) https://BioRender.com/wxrxskk.

The integration of multi-omic data begins at the single-cell level with isolation, molecular barcoding, and sequencing, followed by the application of computational frameworks that align and merge the resulting multi-layered datasets (Figure 2).

#### 2.3.1. Single Cell: Isolation

Single-cell RNA sequencing (scRNA-seq) has become integral to dissecting the heterogeneity of cell populations in the study of wound healing. Techniques such as 10× Genomics Chromium, Drop-seq, and Smart-seq enable the isolation and barcoding of individual cells, allowing for high-throughput sequencing of their transcriptomes. These methods facilitate the identification of distinct cell types and states involved in the healing process. For instance, scRNA-seq has been utilized to analyze epithelial cells during early wound responses, revealing dynamic changes in gene expression that correlate with different healing stages [42].

The process begins with single-cell isolation, where heterogeneous tissue samples (e.g., injured skin or fibrotic muscle) are dissociated into viable, individual cells. Techniques such as Fluorescence-Activated Cell Sorting (FACS) provide high-purity separations based on fluorescently labeled surface markers, although mechanical stress during sorting can perturb cell states [43]. Magnetic-Activated Cell Sorting (MACS), which uses antibody-conjugated magnetic beads, offers a gentler alternative but with lower throughput [44]. Microfluidic droplet systems, including the 10× Genomics Chromium, are widely adopted for their increased capacity to encapsulate thousands of cells into nanoliter droplets compatible with downstream barcoding [45,46]; thus allowing for high-throughput capture of single-cell transcriptomes, and increasingly, chromatin accessibility and protein expression alongside robust scalability. For spatially resolved studies, laser capture microdissection (LCM) allows for excision of cells directly from tissue sections, preserving spatial context but with challenges owing to limiting throughput and the manual nature of the cell excision. Despite these barriers, however, LCM remains critical for integrating spatial transcriptomics or proteomics with histopathological features [47].

#### 2.3.2. Single Cell: Barcoding

Following the physical isolation of individual cells or nuclei, barcoding strategies are used to index each cell’s molecular content. These methods encapsulate individual cells with barcoded gel beads containing oligonucleotides that include poly(dT) sequences for mRNA capture, unique cell-specific barcodes, and Unique Molecular Identifiers (UMIs) [48,49]. The poly(dT) region hybridizes to the poly-A tail of mRNAs, enabling transcript capture, while UMIs serve to mitigate PCR amplification biases by uniquely tagging each mRNA molecule.

Alternative methods such as split-pool combinatorial barcoding (e.g., single cell combinatorial indexing RNA sequencing (sci-RNA-seq), split pool ligation-based transcriptome (SPLiT-seq)) label cells through iterative rounds of barcode assignment, eliminating the need for microfluidics and allowing profiling of millions of cells [50]. This carries the risk for increased potential for barcode collisions, however, which can confound single-cell resolution and require sophisticated computational methods to deconvolute [51,52]. For tissues that are difficult to dissociate or have dissociation-induced transcriptional stress responses, such as fibrotic scars, single-nucleus RNA-seq (snRNA-seq) is employed to analyze nuclear transcripts from frozen samples [53,54].

#### 2.3.3. Single Cell: Sequencing

Sequencing follows, typically using Illumina platforms like NovaSeq, producing vast digital gene expression matrices that quantify transcript abundance across thousands of individual cells [55]. Modern extensions allow for simultaneous measurement of multiple molecular modalities. For example, CITE-seq (Cellular Indexing of Transcriptomes and Epitopes) integrates surface protein quantification via oligo-tagged antibodies with transcriptomics [56]. This enables precise immunophenotyping of immune cells, refining our understanding and identification of cellular states, including macrophage polarization dynamics and T cell activation, in chronic and acute wounds [57,58]. Single-cell trajectory inference tools, including RNA velocity and Monocle 3, further extend these insights by reconstructing lineage relationships and predicting cell fate transitions—crucial for elucidating how progenitor populations contribute to re-epithelialization or stromal remodeling [59,60,61].

scATAC-seq (single-cell assay for transposase-accessible chromatin with high-throughput sequencing) profiles chromatin accessibility, and when integrated with scRNA-seq, offers joint insights into gene regulation [29,62]. This combined approach has elucidated the epigenetic landscapes that underpin cellular responses to injury, offering a more comprehensive understanding of the molecular events driving tissue regeneration.

Multiome platforms such as 10× Genomics’ ATAC + Gene Expression system simultaneously capture transcriptomes and epigenomic data from the same cell [63]. Other tools, like scCUT&Tag, provide chromatin state information by measuring histone modifications, offering a detailed view of the epigenetic landscape during wound healing [64].

Spatial omics technologies are providing the contextual information necessary to map the molecular activity captured through scRNA-seq within the anatomical framework of healing tissue [65]. Spatial transcriptomics platforms such as 10× Visium, Slide-seq, and Multiplexed error-robust fluorescence in situ hybridization (MERFISH) preserve tissue architecture while capturing spatially resolved gene expression profiles, enabling the identification of spatial gradients in inflammatory signaling, revascularization, and epidermal regeneration [66,67].

To integrate these often diverse datasets, computational frameworks such as Seurat v4’s Weighted Nearest Neighbor Analysis, MOFA+, or TotalVI (for RNA-protein integration) are used to align features across modalities [68]. These integrated datasets are indispensable in wound healing research. They have uncovered dynamic immune cell heterogeneity—such as temporal macrophage polarization and neutrophil states—distinct fibroblast subtypes driving fibrosis or regeneration, and spatial gene expression patterns across wound beds when paired with spatial transcriptomics. Furthermore, single-cell multi-omics has elucidated epigenomic priming of cells poised for proliferation or differentiation, and enabled reconstruction of differentiation trajectories via pseudotime analysis [69]. Tools like CellChat, CellPhoneDB, and NicheNet facilitate inference of cell–cell communication networks, revealing ligand–receptor interactions that coordinate tissue repair [70,71,72].

### 2.4. Novel Insights from Multi-Omic Studies in Wound Healing

In a study by Liu et al. [73], scRNA-seq and Visium spatial transcriptomics were conducted on human skin wound tissues collected from the same individuals as they progressed through the inflammatory, proliferative, and remodeling phases of wound repair. This integrative multi-omic approach enabled monitoring of cellular and molecular wound healing dynamics in vivo, culminating in the development of a cellular atlas of human skin wound healing at unprecedented spatiotemporal resolution. The incorporation of CellChat further enhanced this analysis by identifying key ligand–receptor interactions and cell-to-cell communication pathways driving tissue repair. Notably, this analysis achieved a more refined understanding of the role of keratinocytes in wound healing than previously understood. CellChat revealed that keratinocytes actively initiate immune responses by secreting the cytokines interleukin (IL)18, CCL27, and chemokine (C-X-C motif) ligand (CXCL)14—molecules that are often overlooked in wound healing research. Moreover, it challenged in vitro assumptions by demonstrating that cytokines typically attributed to keratinocytes, such as IL 1A/B, IL6, and CXCL1/5/8, are predominantly produced by macrophages, dendritic cells, and fibroblasts in vivo. This underscores the importance of multi-omic technologies in accurately characterizing intercellular communication during wound repair.

CellChat also revealed that CXCL1, produced by pro-inflammatory macrophages, plays a crucial role in promoting keratinocyte migration, challenging the current understanding that attributes it to neutrophil recruitment. Functional validation demonstrated that blocking its receptor, CXCR2, impaired re-epithelialization independent of neutrophils, reinforcing the importance of macrophage-driven inflammation in facilitating tissue repair. Pairing these findings with single-cell data from venous and diabetic foot ulcers established a direct link between failed keratinocyte migration and impaired inflammatory response in chronic wounds. This reframes chronic wound pathology as a problem of immune dysregulation rather than simply excessive inflammation, suggesting that the precise modulation of pathological inflammation, rather than its suppression, may hold the key to restoring healing in chronic wounds. Finally, cross-species comparisons with mouse wound transcriptomes revealed both shared and unique repair mechanisms, emphasizing the atlas’s utility in validating the clinical relevance of findings from animal models, as well as in vitro models. Collectively, this study demonstrates how multi-omics is revolutionizing our understanding of wound healing, while paving the way for more mechanistically informed therapies for chronic wounds.

Multi-omics has also illustrated how fibroblasts transition through distinct functional states during wound healing. In a study by Foster et al. [74], scRNA-seq, ATAC-seq, and Visium spatial transcriptomics were used to map the transcriptional and epigenetic landscape of fibroblasts throughout the course of wound repair. This analysis revealed that fibroblasts are not a homogenous population but instead differentiate into distinct, functionally specialized subtypes in response to injury. Four provisional fibroblast subpopulations were identified—mechano-fibrotic, activated-responder, proliferator, and remodeling—each with spatial and temporal specificity. Mechanofibrotic fibroblasts, localized at the wound margin, expressed high levels of fibrosis-associated genes including Engrailed-1 (En1), Col1a1, TGFβ2, and Jun. These cells emerged early and remained prominent throughout wound maturation. In contrast, proliferator fibroblasts expanded to fill the wound gap, while remodeling fibroblasts appeared later and contributed to ECM reorganization. Activated-responder fibroblasts were detectable as early as postoperative day 2, initiating key regenerative processes. The integration of ATAC-seq data further revealed that upstream changes in chromatin accessibility at mechanosensitive gene loci occurred prior to transcriptional activation, establishing a mechanistic link between tissue tension and fibroblast differentiation.

Clinically, these advances offer multiple opportunities for translation. By identifying mechanical signaling as an upstream driver of fibrosis, this study suggests that targeting pathways such as YAP/TAZ, integrins, or focal adhesion kinase may suppress the early fibrotic programming in mechano-fibrotic fibroblasts. Moreover, the distinct transcriptional signatures of fibroblast subtypes provide a framework for developing cell state–specific therapies that inhibit pro-fibrotic activity while preserving or enhancing the regenerative capacity of remodeling fibroblasts. Importantly, the temporal resolution of the data identifies critical intervention windows—particularly around postoperative days 2 and 7—when fibroblast fate decisions are being determined. Therapies administered within this window could be highly effective in influencing wound outcomes.

Importantly, while these multi-omic technologies excel at revealing mechano-sensitive pathways, fibroblast subpopulations, and regulators of fibrosis, classical genetic knockdown or knockout models remain essential for establishing causality and dissecting mechanistic roles in vivo. Studies on En1-positive fibroblast lineages further exemplify this synergy [75]. Using spatiotemporally resolved lineage tracing to map when and where scarring fibroblasts arise during wound repair, En1-positive fibroblasts were demonstrated to emerge locally within the wound bed through tension-dependent activation of En1 in En1-negative fibroblasts, rather than through simple expansion of embryonic En1-positive cells. Multi-omic profiling further showed that this spatially and temporally restricted transition is accompanied by activation of a YAP-driven profibrotic transcriptional program with increased ECM production. Functional studies confirmed that YAP knockout prevents En1 activation, redirecting fibroblasts toward regenerative healing, restoring hair follicles, glands, and normal ECM architecture. Thus, rather than serving as a replacement for classical genetic models, multi-omic technologies act synergistically with them—guiding hypothesis generation, refining cellular context, and extending the mechanistic insight derived from foundational genetic models that have long driven progress in wound healing research, and will continue to do so.

### 2.5. Challenges and Limitations of Multi-Omic Approaches

#### 2.5.1. Technical and Methodological Challenges

Despite the potential of multi-omic technologies in helping to elucidate the molecular landscape of wound healing, numerous technical and methodological challenges limit their widespread application. One challenge lies in the heterogeneity and instability of wound tissue samples. Wound environments are composed of diverse cell types (e.g., fibroblasts, keratinocytes, endothelial cells, immune cells) and are often colonized by microbes, all of which contribute to highly variable molecular profiles across patients and wound types [76,77]. This complexity is compounded by the dynamic nature of wound healing, where molecular signals change rapidly over time, making it difficult to identify consistent biomarkers unless sampling is rigorously timed and standardized [78]. Furthermore, wound tissue, particularly those in chronic or inflamed conditions, often contains regions of necrosis, ischemia, and high oxidative stress, which promote rapid degradation of biomolecules. RNA, being inherently unstable, is especially vulnerable to degradation by endogenous and exogenous ribonucleases (RNases), which are abundant in inflamed or infected tissues. Inadequate preservation or delayed processing of tissue biopsies can lead to fragmentation of RNA, resulting in poor sequencing coverage, biased gene expression quantification, and compromised transcriptomic data integrity [79].

In wound-healing studies specifically, methodological variability in tissue collection, storage, and processing can further influence downstream analyses [80,81]. In particular, differences in enzymatic digestion methods or temperature can bias cellular composition and distort transcriptomic profiles [81,82]. Variation in experimental endpoints, such as whether analyses are performed at re-epithelialization, granulation, or remodeling stages, may also further impact the comparability of multi-omic datasets across studies.

#### 2.5.2. Data Integration and Interpretation Complexities

Integrating and interpreting the high-dimensional data output of multi-omics presents considerable analytical and conceptual challenges. Multi-omic platforms encompass diverse data types—including genomics, transcriptomics, proteomics, metabolomics, epigenomics, and microbiomics—each with its own experimental noise profile, temporal resolution, dynamic range, and context-dependent biological significance [83]. These differences show the inherent challenge of inter-omic data harmonization. For instance, while genomics data (e.g., SNPs, structural variants) are relatively static, transcriptomic data are transient and dynamic, reflecting acute responses to injury or inflammation [84]. Proteomic data introduce further complexity, as protein abundance is subject to post-transcriptional and post-translational modifications, which often result in weak or inconsistent correlations with corresponding mRNA levels [85,86]. Metabolomic data, which provide snapshots of cellular and extracellular metabolic states, are sensitive to environmental fluctuations, tissue viability, and ex vivo degradation—factors observed in wound infection models with porcine skin and that are compounded by the interpretive challenge when merging datasets from necrotic, ischemic, or inflamed wound environments [87,88,89].

Integration methodologies themselves remain an active area of research. Various mathematical and computational frameworks have been developed to integrate multi-omic layers, including similarity network fusion (SNF), Bayesian network modeling, partial least squares regression (PLSR), multi-table canonical correlation analysis (mtCCA), and machine learning-based strategies (e.g., random forest integration, multi-omic autoencoders) [90,91]. While powerful, these methods are often sensitive to missing data, inter-platform variability, and require sophisticated preprocessing steps such as normalization, batch correction, and dimensionality reduction. Additionally, they may suffer from overfitting or reduced generalizability when applied to small or heterogeneous clinical cohorts, a frequent limitation in wound care studies [92,93,94]. Interpretive frameworks, such as pathway enrichment analysis and network biology, must also contend with incomplete or non-uniform pathway annotations, particularly in understudied tissue types like chronically wounded skin or granulation tissue [95], thus limiting the potential biological insight that can be reliably derived from otherwise statistically significant multi-omic signals.

Adding to this complexity are inter-sample and intra-sample sources of heterogeneity. Biopsies from wound beds may differ in cellular composition (e.g., presence of fibroblasts, keratinocytes, immune cells, or microbial biofilms), tissue oxygenation, and depth of sampling [76]. Such variability introduces batch effects and confounds differential expression or abundance analyses if not properly accounted for. Even advanced statistical correction methods, such as surrogate variable analysis (SVA) or Combat-seq, may struggle when biological signals are entangled with technical or spatial noise [96,97]. These challenges are further magnified when integrating data across different species, as differences in skin architecture, immune responses, fibroblast lineage composition, and healing kinetics limit direct cross-species comparability between commonly used animal models and human wounds [98,99]. For example, mice primarily close their wounds via contraction of the panniculus carnosus, a structure absent in humans [100]. Furthermore, murine wounds contain more fibroblast and endothelial cells while human wounds exhibit greater mast cell abundance and heterogeneity, highlighting species-specific differences in cellular contributors to repair [101]. Moreover, biological heterogeneity, which are driven by patient-specific factors such as age, comorbidities, medications, and wound chronicity, further complicates the generalization of -omic findings across populations [102].

Finally, the interpretation of integrated multi-omic results necessitates high-level domain expertise and robust visualization tools to prioritize biologically meaningful patterns. Interpreting thousands of molecular features across data types requires knowledge of gene function, pathway topology, and cellular physiology in the wound microenvironment. Without such contextual understanding, there is a risk of overinterpreting spurious associations or failing to recognize clinically relevant biomarkers [103,104]. The emergence of standardized wound tissue processing pipelines and cross-platform reference atlases or open-access wound healing databases may help address some of these limitations by improving reproducibility, comparability, and translation of multi-omic findings into mechanistic and clinical insight. This underscores the importance of multidisciplinary collaboration—among bioinformaticians, systems biologists, wound care clinicians, and pathologists, for example—in both study design and data interpretation.

#### 2.5.3. Cost-Effectiveness and Accessibility in Clinical Settings

The translation of multi-omic technologies into clinical wound healing practice is fundamentally constrained by significant cost-effectiveness and accessibility challenges. Multi-omic platforms demand substantial capital investment in high-throughput sequencing machines, mass spectrometry equipment, and advanced laboratory infrastructure, coupled with recurrent costs for reagents, consumables, and maintenance [103,105]. Moreover, these methodologies require specialized personnel with expertise in molecular biology, bioinformatics, and computational analysis, which adds layers of operational complexity and financial burden [78]. Additionally, the logistical demands of multi-omic workflows—including stringent sample handling, long processing times, and data-intensive analysis—pose substantial barriers to rapid clinical application, especially in acute wound care where timely intervention is critical [106,107].

Beyond direct financial and infrastructural constraints, the broader clinical integration of multi-omics is hindered by the lack of standardized reimbursement frameworks and insufficient evidence-based guidelines and regulations in precision-based, personalized medicine, in general [108]. The complexity and volume of multi-omic data necessitate advanced integrative analytics and computational resources that are often beyond the scope of routine hospital settings, limiting the interpretability and application of these approaches in clinical workflows [109]. To mitigate these challenges, efforts are underway to develop targeted multi-omic panels focusing on elucidating those clinically actionable biomarkers specific to wound healing pathophysiology, wherein existing technologies in related biomedical fields could offer a foundation for leveraging future research and clinical applications in wound care [110,111,112,113]. In time, interdisciplinary collaboration among clinicians, bioinformaticians, health economists, and policy makers is essential to align technological innovation with pragmatic clinical and economic realities, thereby fostering equitable and cost-effective implementation of multi-omic tools in precision wound management.

### 2.6. Future Directions

#### 2.6.1. Integration of Artificial Intelligence and Machine Learning

**Figure 3:**
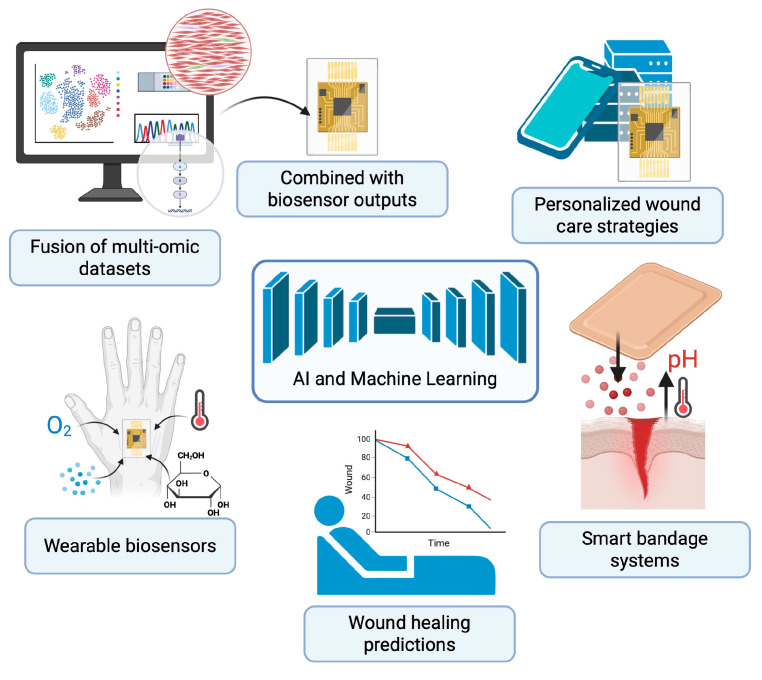
Future of Wound Healing Using Multi-Omic Technologies. Integration of multi-omics, AI, and wearable biosensors is transforming wound care toward predictive, personalized, and adaptive strategies. This schematic demonstrates the fusion of multi-omic datasets with real-time outputs from wearable biosensors to guide personalized wound care strategies. Smart bandage systems continuously monitor wound parameters such as pH, temperature, oxygenation, metabolites, and cytokines, while delivering targeted therapies in response to changes in wound microenvironment. AI-driven analysis of fused multi-omic and sensor data enables predictive modeling of healing outcomes, supporting precision interventions tailored to individual patients. Created in BioRender. Huang, K. (2025) https://BioRender.com/wxrxskk.

The integration of AI and machine learning (ML) into wound healing research is catalyzing a shift in how complex biological data is interpreted and applied clinically (Figure 3). Modern deep learning frameworks such as variational autoencoders (e.g., scVI, totalVI), graph neural networks, and multimodal factor analysis tools (e.g., MOFA+) are now enabling the fusion of diverse -omic datasets—transcriptomic, proteomic, spatial, and imaging-based—while preserving cellular and spatial heterogeneity [114,115]. These methods have been used to identify fibroblast and immune cell subpopulations critical to matrix remodeling and inflammation resolution, providing deeper insight into molecular determinants of healing trajectories. Studies have successfully applied graph neural networks and other ML algorithms to integrate transcriptomic and histological imaging data, uncovering key regenerative cellular circuits in murine wound models [116,117].

AI models are also being developed for predictive analytics, aiming to forecast wound healing outcomes based on both clinical and molecular variables. Supervised learning techniques, including support vector machines and gradient-boosted trees, are being trained on multi-omic and electronic health record (EHR) data to stratify patients into healing risk categories and predict chronicity [118,119]. Studies have demonstrated that time-series machine learning models could predict non-healing diabetic ulcers four weeks in advance with high accuracy and higher AUROC scores than other statistical predictive models [120]. In parallel, the concept of the “digital twin”—a computationally dynamic and biologically personalized model of a patient’s wound—is gaining traction [121,122,123]. These in silico frameworks integrate omic inputs with mechanistic wound models to simulate healing responses under various interventions, potentially guiding personalized therapy in real-time. A recent example is the Wound Environment Agent-Based Model (WEABM), which combines transcriptomic and immunologic data to simulate and characterize volumetric tissue regeneration under experimental treatments [124].

#### 2.6.2. Development of Wearable Biosensors and Continuous Monitoring Technologies

Traditional wound evaluation methods—such as visual inspections or laboratory testing—are often intermittent and subjective, limiting timely interventions [106,125]. In contrast, wearable biosensors offer continuous monitoring of critical physiological parameters such as pH, temperature, oxygenation, glucose, and specific cytokines [126,127]. These indicators are essential in assessing inflammation, infection, tissue perfusion, and metabolic activity in chronic wounds. As wound pathophysiology is inherently dynamic, biosensors provide clinicians with an ongoing stream of objective data, potentially leading to faster diagnosis of complications and improved treatment speed, with an additional emphasis on the potential for these biosensors to improve patient outcomes and reduce healthcare burdens [128].

Emerging smart bandage systems integrate flexible electronics with responsive materials to not only sense wound status but also deliver therapeutics in a feedback-controlled manner [129]. These smart dressings can, for example, release antibiotics when an infection is detected via elevated pH or temperature levels [130,131]. Some platforms incorporate wireless telemetry for potential remote monitoring, allowing caregivers or healthcare providers to track wound progress without requiring frequent clinic visits [129,131]. This is particularly valuable in managing chronic wounds in diabetic or immobile patients, where early signs of deterioration can be subtle yet clinically significant [132,133]. Moreover, the integration of biosensors with smartphone applications and cloud-based systems enhances patient engagement and enables more personalized wound care strategies.

Beyond real-time sensing, a major innovation lies in the convergence of biosensor outputs with multi-omic data to create a systems-level understanding of wound healing [134]. For instance, biosensor-detected fluctuations in cytokines or metabolites can be cross-referenced with transcriptomic, proteomic, or microbiomic profiles to identify biomarkers associated with healing or chronicity [106,135]. Current insights into the molecular mechanism of cancer progression and novel early biomarkers detection by multi-omics approaches are the key to tackling tumor and metastases progression via biosensor based diagnosis of biomarkers [136].

Despite this potential, challenges remain in translating these technologies into widespread clinical use. Key limitations include ensuring biocompatibility and functionality of biosensors in the harsh, protease-rich wound environment [137]; developing energy-efficient systems for continuous use [138]; standardizing data interpretation across platforms; and the aforementioned addressing of cost-effectiveness for scalability in various healthcare settings [78]. Regulatory approval processes and integration with existing electronic health record systems also need refinement, while also ensuring more precision-guided wound management—moving away from one-size-fits-all interventions toward personalized care plans.

Nonetheless, as biosensor technology continues to mature and multi-omic analytics become more accessible, the next decade is poised to witness a significant leap in smart wound care, underpinned by continuous monitoring and personalized treatment.

## 3. Conclusions

The integration of multi-omic technologies into wound healing research has catapulted our understanding of both insufficient and excessive healing, revealing the intricate cellular interactions and molecular pathways that govern these divergent outcomes. The rich, multi-layered insights generated through multi-omics have not only revealed critical biomarkers and dysfunctional cellular crosstalk but also challenged existing paradigms of wound biology, opening new avenues for targeted intervention.

Multi-omic biomarkers offer the potential to identify wounds at risk of chronicity early, while mechanistic insights guide the development of therapies that modulate specific cell states or signaling pathways. In addition, spatially resolved -omic data lay the groundwork for the design of advanced wound biomaterials capable of directing local immune or stromal responses to favor regeneration. Paired with emerging biosensor technologies, these tools enable a shift toward more personalized and timely wound care.

Despite challenges in data integration, clinical translation, and scalability, multi-omic technologies are rapidly reshaping the landscape of wound healing and care. Fully harnessing its potential will require the continued advancement of tools capable of translating complex molecular insights into precise diagnostics and targeted therapies, advancing us closer to the prospect of true tissue regeneration, once thought to be beyond reach.

## Figures and Tables

**Table 1:** Summary of multi-omic contributions to wound healing. Insights into specific wound healing phases, from genetic susceptibility to dynamic changes in gene, protein, and metabolite expression.

-Omic Technology	Key Phases of Wound Healing	Insights
Genomics	Inflammation, Proliferation, Remodeling	Variants in TLN2 and ZNF521 linked to infection susceptibility [18] CSMD1 regulates complement inhibition and TGF-β/SMAD signaling across phases [19]
Proteomics	Inflammation, Proliferation, Remodeling	Elevated S100A9 in chronic wounds [23] Collagen I/III, tetranectin, and SERPINs enriched in healing wounds, Fibronectin, vitronectin dominate in stalled wounds [24]
Transcriptomics	Hemostasis, Inflammation, Proliferation, Remodeling	Variable transcriptional patterns during hemostasis and early inflammation across tissue type and injury context [27] YAP, TAZ, Piezo1, RhoA, and ROCK2 upregulated in keloid fibroblasts [26] Increased VEGFA, COL1A1, and MMP12 increased during later phases of wound healing [27] Immune gene expression persists beyond inflammation [27]
Epigenomics	Proliferation, Remodeling	Persistent THBS1-accessibility in irradiated fibroblasts [35,36] PcG-mediated gene silencing controls epithelial migration and remodeling [34] DNMT1 regulates keratinocyte proliferation with its loss impairing re-epithelialization [35] Fibroblast methylation changes (RUNX2, α-SMA) drive fibrosis [35,36]
Metabolomics	Inflammation, Proliferation, Remodeling	Linoleic acid suppresses inflammation [39] D-(+)-galactose and glycerol promote ECM remodeling and re-epithelialization [40]

## Data Availability

No new data were created or analyzed in this study. Data sharing is not applicable to this article.

## Abbreviations

The following abbreviations are used in this manuscript:

AI	Artificial Intelligence
PDGF	Platelet-Derived Growth Factor
TGF-β	Transforming Growth Factor Beta
ECM	Extracellular Matrix
VEGF	Vascular Endothelial Growth Factor
FGF	Fibroblast Growth Factor
MMPs	Matrix Metalloproteases
TIMPs	Tissue Inhibitors of Metalloproteases
GWAS	Genome-Wide Association Studies
TLN2	Talin-2
ZNF521	Zinc Finger Protein 521
CSMD1	CUB and Sushi Multiple Domains 1 Gene
SERPIN	Serine Protease Inhibitor
RNA-seq	RNA Sequencing
scRNA-seq	Single-Cell RNA Sequencing
YAP	Yes-Associated Protein
TAZ	Tafazzin
Piezo1	Piezo-Type Mechanosensitive Ion Channel Component 1
RhoA	Ras Homolog Family Member A
ROCK2	Rho-Associated Protein Kinase 2
En1	Engrailed-1
COL1A1	Collagen Type I Alpha 1 Chain
CCL2	CC Motif Chemokine Ligand 2
ATAC-seq	Assay for Transposase-Accessible Chromatin with High-Throughput Sequencing
THBS1	Thrombospondin 1
ChIP-seq	Chromatin Immunoprecipitation Sequencing
PcG	Polycomb Group
DNMT1	DNA Methyltransferase 1
RUNX2	Runt-Related Transcription Factor 2
α-SMA	Alpha-Smooth Muscle Actin
NMR	Nuclear Magnetic Resonance Spectroscopy
TNF-α	Tumor Necrosis Factor Alpha
IL-12	Interleukin-12
FACS	Fluorescence-Activated Cell Sorting
MACS	Magnetic-Activated Cell Sorting
LCM	Laser Capture Microdissection
UMIs	Unique Molecular Identifiers
sci-RNA-seq	Single Cell Combinatorial Indexing RNA Sequencing
SPLiT-seq	Split Pool Ligation-Based Transcriptome Sequencing
snRNA-seq	Single-Nucleus RNA Sequencing
CITE-seq	Cellular Indexing of Transcriptomes and Epitopes
scATAC-seq	Single-Cell Assay for Transposase-Accessible Chromatin with High-Throughput Sequencing
MERFISH	Multiplexed Error-Robust Fluorescence In Situ Hybridization
CXCL14	C-X-C Motif Ligand 14
SNF	Similarity Network Fusion
PLSR	Partial Least Squares Regression
mtCCA	Multi-Table Canonical Correlation Analysis
SVA	Surrogate Variable Analysis
ML	Machine Learning
EHR	Electronic Health Record
WEABM	Wound Environment Agent-Based Model
